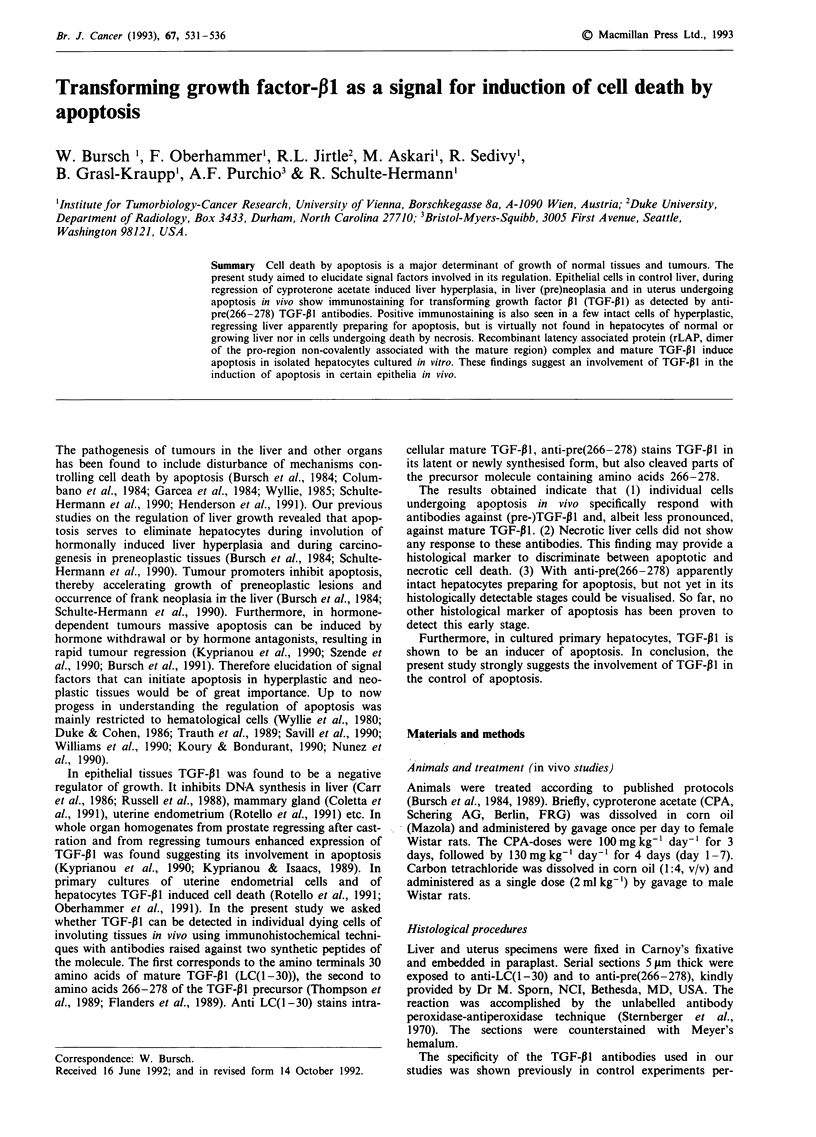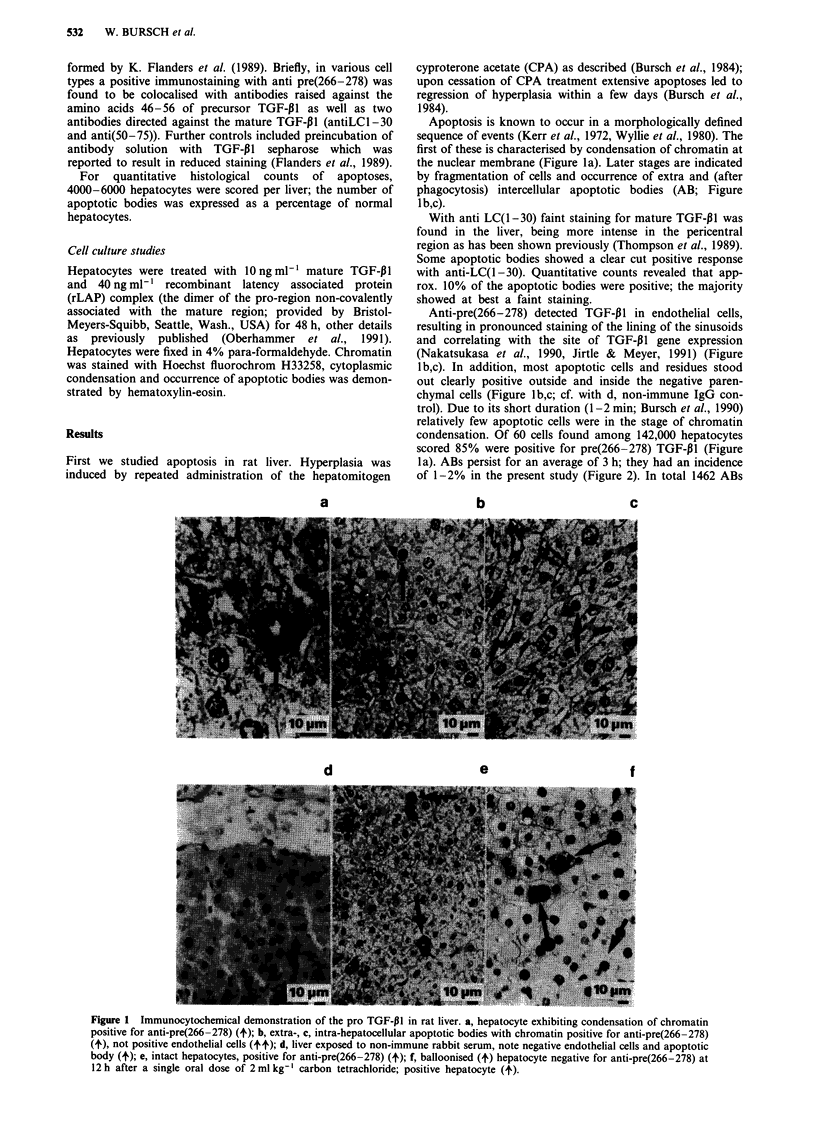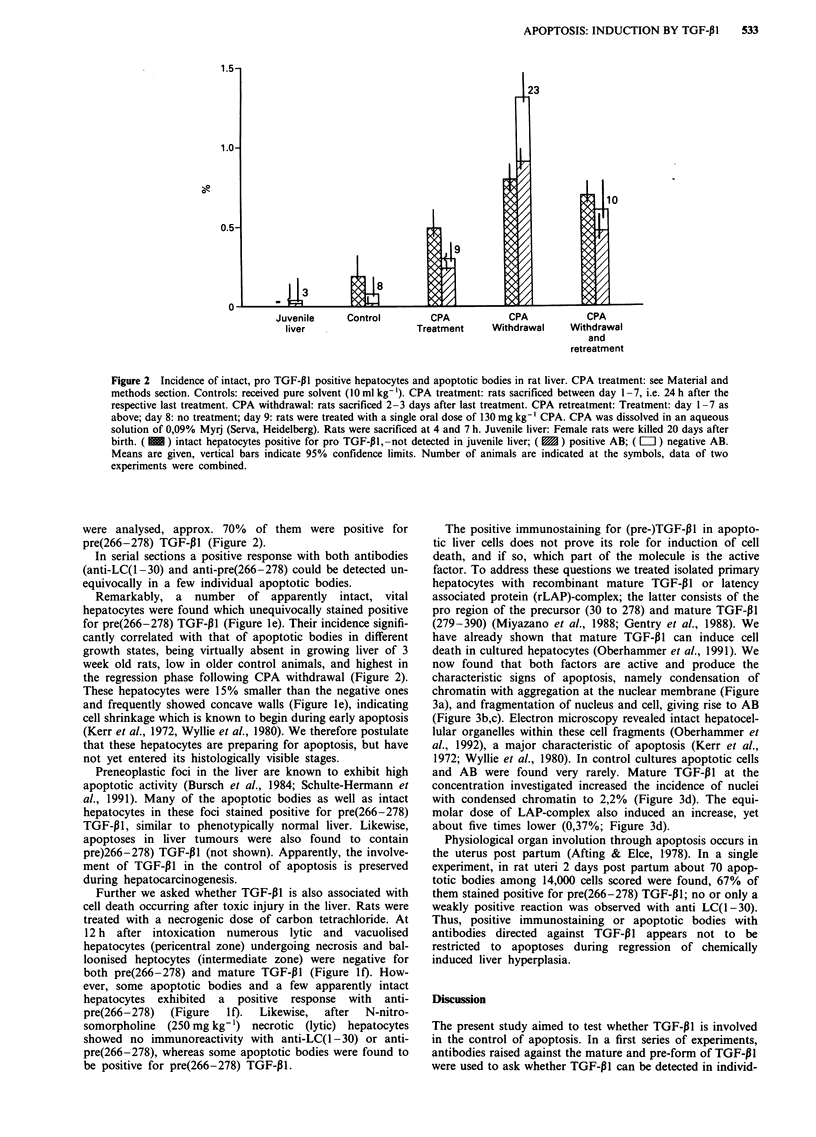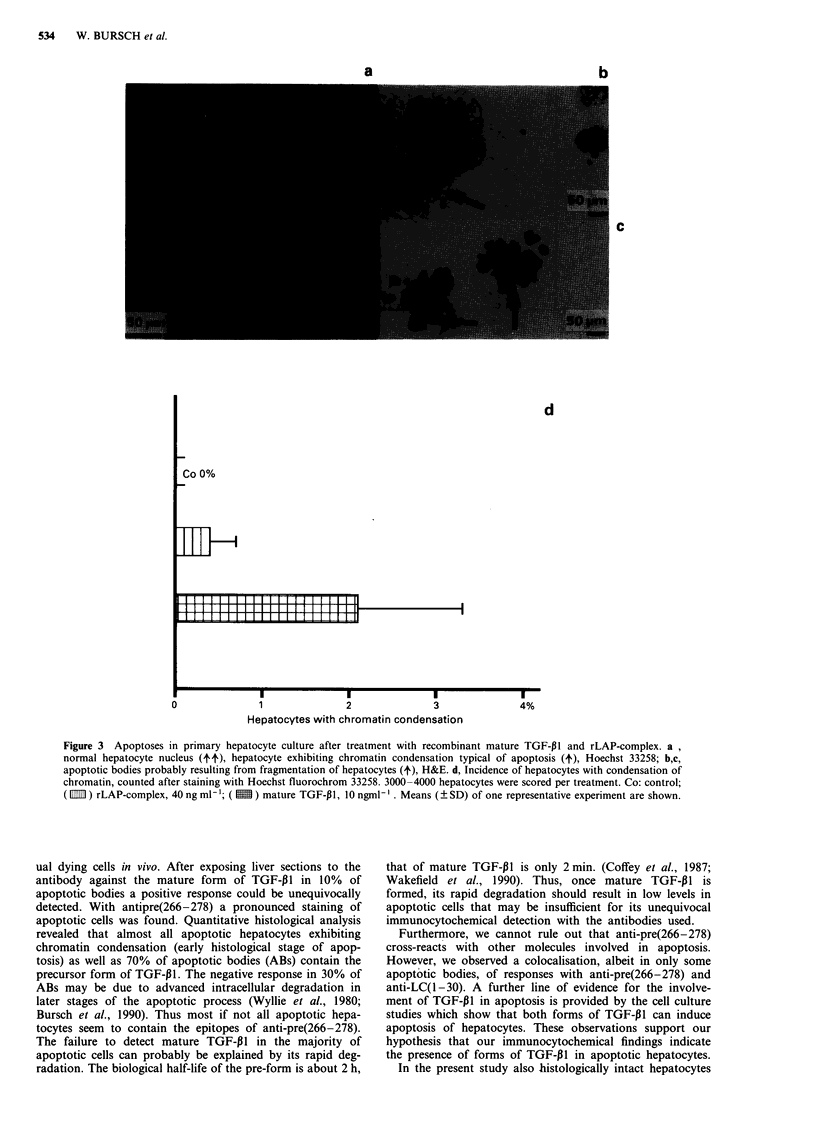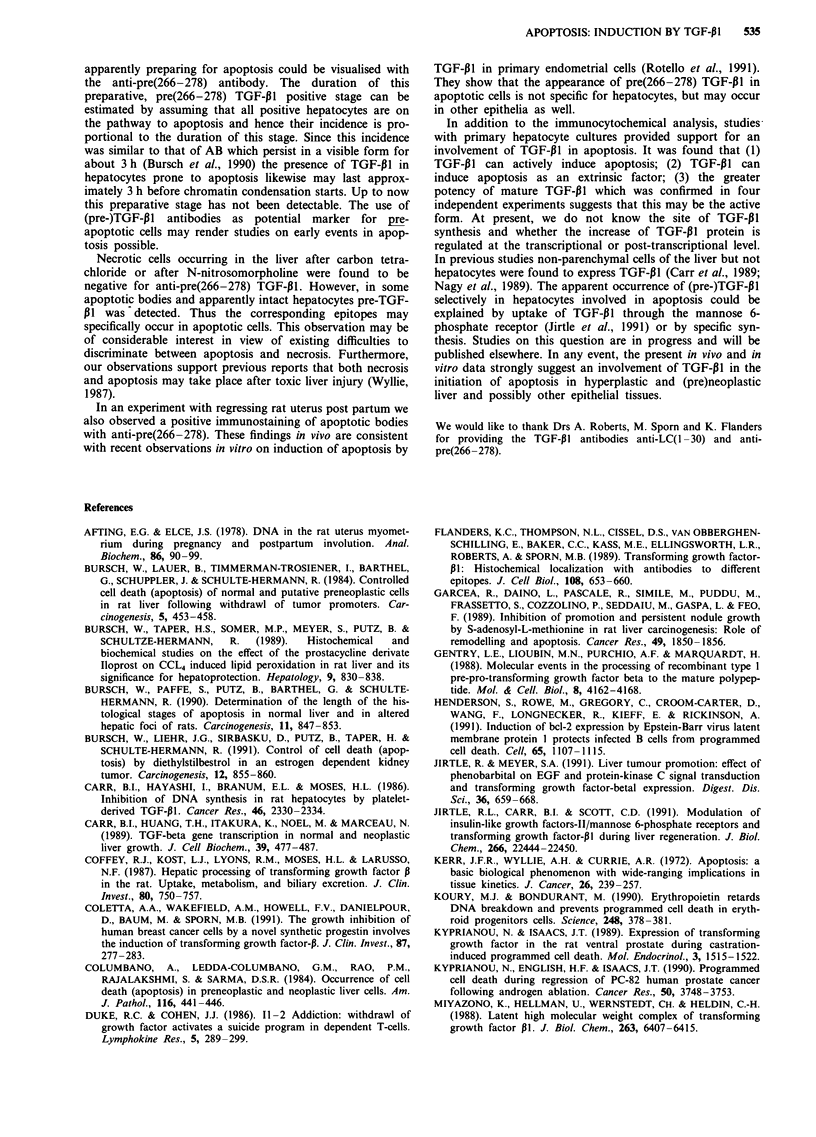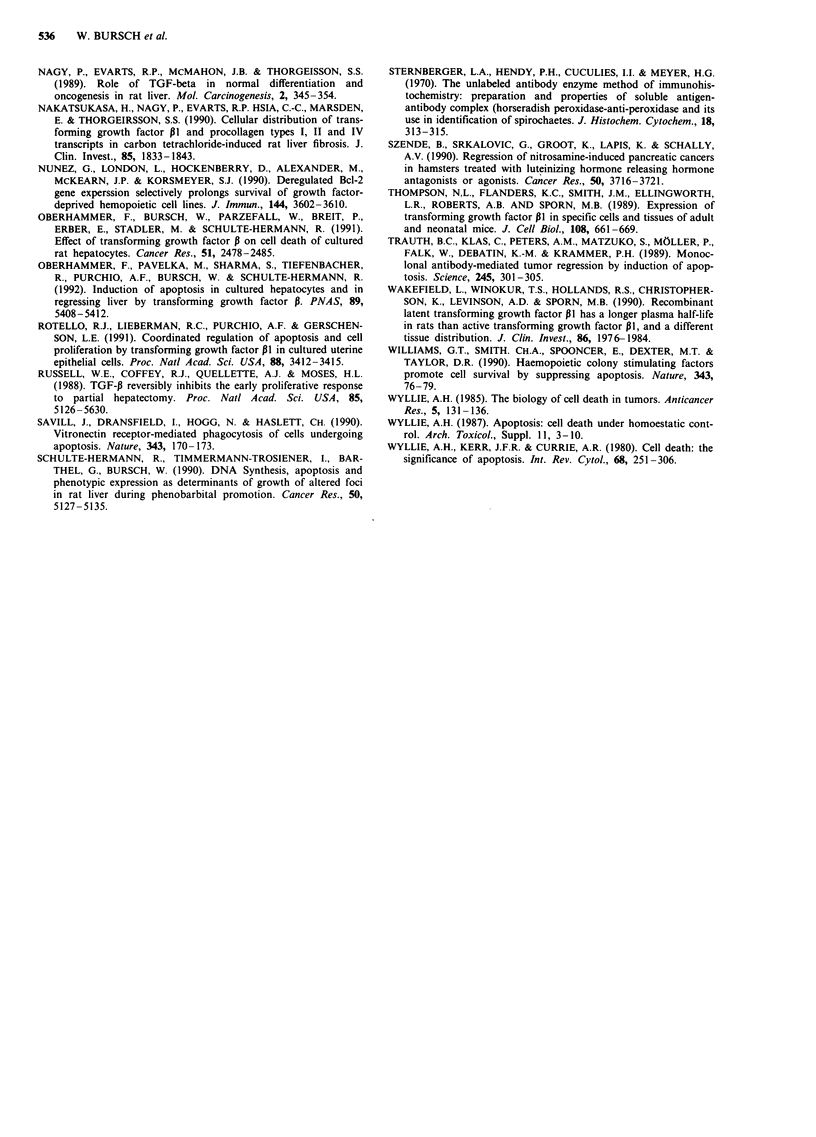# Transforming growth factor-beta 1 as a signal for induction of cell death by apoptosis.

**DOI:** 10.1038/bjc.1993.98

**Published:** 1993-03

**Authors:** W. Bursch, F. Oberhammer, R. L. Jirtle, M. Askari, R. Sedivy, B. Grasl-Kraupp, A. F. Purchio, R. Schulte-Hermann

**Affiliations:** Institute for Tumorbiology-Cancer Research, University of Vienna, Wien, Austria.

## Abstract

**Images:**


*Br. J. Cancer* (1993), **67**, 531–536 © Macmillan Press Ltd., 1993

### Transforming growth factor-β1 as a signal for induction of cell death by apoptosis

W. Bursch [1], F. Oberhammer[1], R.L. Jirtle[2], M. Askari[1], R. Sedivy[1], B. Grasl-Kraupp[1], A.F. Purchio[3] & R. Schulte-Hermann[1]

[1]*Institute for Tumorbiology-Cancer Research, University of Vienna, Borschkegasse 8a, A-1090 Wien, Austria;* [2]*Duke University, Department of Radiology, Box 3433, Durham, North Carolina 27710;* [3]*Bristol-Myers-Squibb, 3005 First Avenue, Seattle, Washington 98121, USA.*

**Summary** Cell death by apoptosis is a major determinant of growth of normal tissues and tumours. The present study aimed to elucidate signal factors involved in its regulation. Epithelial cells in control liver, during regression of cyproterone acetate induced liver hyperplasia, in liver (pre)neoplasia and in uterus undergoing apoptosis *in vivo* show immunostaining for transforming growth factor β1 (TGF-β1) as detected by anti-pre(266–278) TGF-β1 antibodies. Positive immunostaining is also seen in a few intact cells of hyperplastic, regressing liver apparently preparing for apoptosis, but is virtually not found in hepatocytes of normal or growing liver nor in cells undergoing death by necrosis. Recombinant latency associated protein (rLAP, dimer of the pro-region non-covalently associated with the mature region) complex and mature TGF-β1 induce apoptosis in isolated hepatocytes cultured *in vitro*. These findings suggest an involvement of TGF-β1 in the induction of apoptosis in certain epithelia *in vivo*.

The pathogenesis of tumours in the liver and other organs has been found to include disturbance of mechanisms controlling cell death by apoptosis (Bursch *et al.*, 1984; Columbano *et al.*, 1984; Garcea *et al.*, 1984; Wyllie, 1985; Schulte-Hermann *et al.*, 1990; Henderson *et al.*, 1991). Our previous studies on the regulation of liver growth revealed that apoptosis serves to eliminate hepatocytes during involution of hormonally induced liver hyperplasia and during carcinogenesis in preneoplastic tissues (Bursch *et al.*, 1984; Schulte-Hermann *et al.*, 1990). Tumour promoters inhibit apoptosis, thereby accelerating growth of preneoplastic lesions and occurrence of frank neoplasia in the liver (Bursch *et al.*, 1984; Schulte-Hermann *et al.*, 1990). Furthermore, in hormone-dependent tumours massive apoptosis can be induced by hormone withdrawal or by hormone antagonists, resulting in rapid tumour regression (Kyprianou *et al.*, 1990; Szende *et al.*, 1990; Bursch *et al.*, 1991). Therefore elucidation of signal factors that can initiate apoptosis in hyperplastic and neoplastic tissues would be of great importance. Up to now progess in understanding the regulation of apoptosis was mainly restricted to hematological cells (Wyllie *et al.*, 1980; Duke & Cohen, 1986; Trauth *et al.*, 1989; Savill *et al.*, 1990; Williams *et al.*, 1990; Koury & Bondurant, 1990; Nunez *et al.*, 1990).

In epithelial tissues TGF-β1 was found to be a negative regulator of growth. It inhibits DNA synthesis in liver (Carr *et al.*, 1986; Russell *et al.*, 1988), mammary gland (Coletta *et al.*, 1991), uterine endometrium (Rotello *et al.*, 1991) etc. In whole organ homogenates from prostate regressing after castration and from regressing tumours enhanced expression of TGF-β1 was found suggesting its involvement in apoptosis (Kyprianou *et al.*, 1990; Kyprianou & Isaacs, 1989). In primary cultures of uterine endometrial cells and of hepatocytes TGF-β1 induced cell death (Rotello *et al.*, 1991; Oberhammer *et al.*, 1991). In the present study we asked whether TGF-β1 can be detected in individual dying cells of involuting tissues *in vivo* using immunohistochemical techniques with antibodies raised against two synthetic peptides of the molecule. The first corresponds to the amino terminals 30 amino acids of mature TGF-β1 (LC(1–30)), the second to amino acids 266–278 of the TGF-β1 precursor (Thompson *et al.*, 1989; Flanders *et al.*, 1989). Anti LC(1–30) stains intracellular mature TGF-β1, anti-pre(266–278) stains TGF-β1 in its latent or newly synthesised form, but also cleaved parts of the precursor molecule containing amino acids 266–278.

The results obtained indicate that (1) individual cells undergoing apoptosis *in vivo* specifically respond with antibodies against (pre-)TGF-β1 and, albeit less pronounced, against mature TGF-β1. (2) Necrotic liver cells did not show any response to these antibodies. This finding may provide a histological marker to discriminate between apoptotic and necrotic cell death. (3) With anti-pre(266–278) apparently intact hepatocytes preparing for apoptosis, but not yet in its histologically detectable stages could be visualised. So far, no other histological marker of apoptosis has been proven to detect this early stage.

Furthermore, in cultured primary hepatocytes, TGF-β1 is shown to be an inducer of apoptosis. In conclusion, the present study strongly suggests the involvement of TGF-β1 in the control of apoptosis.

### Materials and methods

### *Animals and treatment (*in vivo *studies)*

Animals were treated according to published protocols (Bursch *et al.*, 1984, 1989). Briefly, cyproterone acetate (CPA, Schering AG, Berlin, FRG) was dissolved in corn oil (Mazola) and administered by gavage once per day to female Wistar rats. The CPA-doses were 100 mg $kg^{-1}$ $day^{-1}$ for 3 days, followed by 130 mg $kg^{-1}$ $day^{-1}$ for 4 days (day 1–7). Carbon tetrachloride was dissolved in corn oil (1:4, v/v) and administered as a single dose (2 ml $kg^{-1}$) by gavage to male Wistar rats.

### *Histological procedures*

Liver and uterus specimens were fixed in Carnoy's fixative and embedded in paraplast. Serial sections 5 µm thick were exposed to anti-LC(1–30) and to anti-pre(266–278), kindly provided by Dr M. Sporn, NCI, Bethesda, MD, USA. The reaction was accomplished by the unlabelled antibody peroxidase-antiperoxidase technique (Sternberger *et al.*, 1970). The sections were counterstained with Meyer's hemalum.

The specificity of the TGF-β1 antibodies used in our studies was shown previously in control experiments per-

Correspondence: W. Bursch.
Received 16 June 1992; and in revised form 14 October 1992.

formed by K. Flanders et al. (1989). Briefly, in various cell types a positive immunostaining with anti pre(266–278) was found to be colocalised with antibodies raised against the amino acids 46–56 of precursor TGF-$\beta$1 as well as two antibodies directed against the mature TGF-$\beta$1 (antiLC1–30 and anti(50–75)). Further controls included preincubation of antibody solution with TGF-$\beta$1 sepharose which was reported to result in reduced staining (Flanders et al., 1989).

For quantitative histological counts of apoptoses, 4000–6000 hepatocytes were scored per liver; the number of apoptotic bodies was expressed as a percentage of normal hepatocytes.

*Cell culture studies*

Hepatocytes were treated with 10 ng $ml^{-1}$ mature TGF-$\beta$1 and 40 ng $ml^{-1}$ recombinant latency associated protein (rLAP) complex (the dimer of the pro-region non-covalently associated with the mature region; provided by Bristol-Meyers-Squibb, Seattle, Wash., USA) for 48 h, other details as previously published (Oberhammer et al., 1991). Hepatocytes were fixed in 4% para-formaldehyde. Chromatin was stained with Hoechst fluorochrom H33258, cytoplasmic condensation and occurrence of apoptotic bodies was demonstrated by hematoxylin-eosin.

### Results

First we studied apoptosis in rat liver. Hyperplasia was induced by repeated administration of the hepatomitogen cyproterone acetate (CPA) as described (Bursch et al., 1984); upon cessation of CPA treatment extensive apoptoses led to regression of hyperplasia within a few days (Bursch et al., 1984).

Apoptosis is known to occur in a morphologically defined sequence of events (Kerr et al., 1972, Wyllie et al., 1980). The first of these is characterised by condensation of chromatin at the nuclear membrane (Figure 1a). Later stages are indicated by fragmentation of cells and occurrence of extra and (after phagocytosis) intercellular apoptotic bodies (AB; Figure 1b,c).

With anti LC(1–30) faint staining for mature TGF-$\beta$1 was found in the liver, being more intense in the pericentral region as has been shown previously (Thompson et al., 1989). Some apoptotic bodies showed a clear cut positive response with anti-LC(1–30). Quantitative counts revealed that approx. 10% of the apoptotic bodies were positive; the majority showed at best a faint staining.

Anti-pre(266–278) detected TGF-$\beta$1 in endothelial cells, resulting in pronounced staining of the lining of the sinusoids and correlating with the site of TGF-$\beta$1 gene expression (Nakatsukasa et al., 1990, Jirtle & Meyer, 1991) (Figure 1b,c). In addition, most apoptotic cells and residues stood out clearly positive outside and inside the negative parenchymal cells (Figure 1b,c; cf. with d, non-immune IgG control). Due to its short duration (1–2 min; Bursch et al., 1990) relatively few apoptotic cells were in the stage of chromatin condensation. Of 60 cells found among 142,000 hepatocytes scored 85% were positive for pre(266–278) TGF-$\beta$1 (Figure 1a). ABs persist for an average of 3 h; they had an incidence of 1–2% in the present study (Figure 2). In total 1462 ABs

**Figure 1** Immunocytochemical demonstration of the pro TGF-$\beta$1 in rat liver. **a**, hepatocyte exhibiting condensation of chromatin positive for anti-pre(266–278) (↑); **b**, extra-, **c**, intra-hepatocellular apoptotic bodies with chromatin positive for anti-pre(266–278) (↑), not positive endothelial cells (↑↑); **d**, liver exposed to non-immune rabbit serum, note negative endothelial cells and apoptotic body (↑); **e**, intact hepatocytes, positive for anti-pre(266–278) (↑); **f**, balloonised (↑) hepatocyte negative for anti-pre(266–278) at 12 h after a single oral dose of 2 ml $kg^{-1}$ carbon tetrachloride; positive hepatocyte (↑).

**Figure 2** Incidence of intact, pro TGF-β1 positive hepatocytes and apoptotic bodies in rat liver. CPA treatment: see Material and methods section. Controls: received pure solvent (10 ml $kg^{-1}$). CPA treatment: rats sacrificed between day 1–7, i.e. 24 h after the respective last treatment. CPA withdrawal: rats sacrificed 2–3 days after last treatment. CPA retreatment: Treatment: day 1–7 as above; day 8: no treatment; day 9: rats were treated with a single oral dose of 130 mg $kg^{-1}$ CPA. CPA was dissolved in an aqueous solution of 0,09% Myrj (Serva, Heidelberg). Rats were sacrificed at 4 and 7 h. Juvenile liver: Female rats were killed 20 days after birth. ( ▩ ) intact hepatocytes positive for pro TGF-β1,–not detected in juvenile liver; ( ▨ ) positive AB; ( □ ) negative AB. Means are given, vertical bars indicate 95% confidence limits. Number of animals are indicated at the symbols, data of two experiments were combined.

were analysed, approx. 70% of them were positive for pre(266–278) TGF-β1 (Figure 2).

In serial sections a positive response with both antibodies (anti-LC(1–30) and anti-pre(266–278) could be detected unequivocally in a few individual apoptotic bodies.

Remarkably, a number of apparently intact, vital hepatocytes were found which unequivocally stained positive for pre(266–278) TGF-β1 (Figure 1e). Their incidence significantly correlated with that of apoptotic bodies in different growth states, being virtually absent in growing liver of 3 week old rats, low in older control animals, and highest in the regression phase following CPA withdrawal (Figure 2). These hepatocytes were 15% smaller than the negative ones and frequently showed concave walls (Figure 1e), indicating cell shrinkage which is known to begin during early apoptosis (Kerr *et al.*, 1972, Wyllie *et al.*, 1980). We therefore postulate that these hepatocytes are preparing for apoptosis, but have not yet entered its histologically visible stages.

Preneoplastic foci in the liver are known to exhibit high apoptotic activity (Bursch *et al.*, 1984; Schulte-Hermann *et al.*, 1991). Many of the apoptotic bodies as well as intact hepatocytes in these foci stained positive for pre(266–278) TGF-β1, similar to phenotypically normal liver. Likewise, apoptoses in liver tumours were also found to contain pre)266–278) TGF-β1 (not shown). Apparently, the involvement of TGF-β1 in the control of apoptosis is preserved during hepatocarcinogenesis.

Further we asked whether TGF-β1 is also associated with cell death occurring after toxic injury in the liver. Rats were treated with a necrogenic dose of carbon tetrachloride. At 12 h after intoxication numerous lytic and vacuolised hepatocytes (pericentral zone) undergoing necrosis and balloonised heptocytes (intermediate zone) were negative for both pre(266–278) and mature TGF-β1 (Figure 1f). However, some apoptotic bodies and a few apparently intact hepatocytes exhibited a positive response with anti-pre(266–278) (Figure 1f). Likewise, after N-nitrosomorpholine (250 mg $kg^{-1}$) necrotic (lytic) hepatocytes showed no immunoreactivity with anti-LC(1–30) or anti-pre(266–278), whereas some apoptotic bodies were found to be positive for pre(266–278) TGF-β1.

The positive immunostaining for (pre-)TGF-β1 in apoptotic liver cells does not prove its role for induction of cell death, and if so, which part of the molecule is the active factor. To address these questions we treated isolated primary hepatocytes with recombinant mature TGF-β1 or latency associated protein (rLAP)-complex; the latter consists of the pro region of the precursor (30 to 278) and mature TGF-β1 (279–390) (Miyazano *et al.*, 1988; Gentry *et al.*, 1988). We have already shown that mature TGF-β1 can induce cell death in cultured hepatocytes (Oberhammer *et al.*, 1991). We now found that both factors are active and produce the characteristic signs of apoptosis, namely condensation of chromatin with aggregation at the nuclear membrane (Figure 3a), and fragmentation of nucleus and cell, giving rise to AB (Figure 3b,c). Electron microscopy revealed intact hepatocellular organelles within these cell fragments (Oberhammer *et al.*, 1992), a major characteristic of apoptosis (Kerr *et al.*, 1972; Wyllie *et al.*, 1980). In control cultures apoptotic cells and AB were found very rarely. Mature TGF-β1 at the concentration investigated increased the incidence of nuclei with condensed chromatin to 2,2% (Figure 3d). The equimolar dose of LAP-complex also induced an increase, yet about five times lower (0,37%; Figure 3d).

Physiological organ involution through apoptosis occurs in the uterus post partum (Afting & Elce, 1978). In a single experiment, in rat uteri 2 days post partum about 70 apoptotic bodies among 14,000 cells scored were found, 67% of them stained positive for pre(266–278) TGF-β1; no or only a weakly positive reaction was observed with anti LC(1–30). Thus, positive immunostaining or apoptotic bodies with antibodies directed against TGF-β1 appears not to be restricted to apoptoses during regression of chemically induced liver hyperplasia.

### Discussion

The present study aimed to test whether TGF-β1 is involved in the control of apoptosis. In a first series of experiments, antibodies raised against the mature and pre-form of TGF-β1 were used to ask whether TGF-β1 can be detected in individ-

**Figure 3** Apoptoses in primary hepatocyte culture after treatment with recombinant mature TGF-$\beta$1 and rLAP-complex. **a**, normal hepatocyte nucleus (↑↑), hepatocyte exhibiting chromatin condensation typical of apoptosis (↑), Hoechst 33258; **b,c**, apoptotic bodies probably resulting from fragmentation of hepatocytes (↑), H&E. **d**, Incidence of hepatocytes with condensation of chromatin, counted after staining with Hoechst fluorochrom 33258. 3000–4000 hepatocytes were scored per treatment. Co: control; (▥) rLAP-complex, 40 ng ml$^{-1}$; (▦) mature TGF-$\beta$1, 10 ngml$^{-1}$. Means ($\pm$SD) of one representative experiment are shown.

ual dying cells *in vivo*. After exposing liver sections to the antibody against the mature form of TGF-$\beta$1 in 10% of apoptotic bodies a positive response could be unequivocally detected. With antipre(266–278) a pronounced staining of apoptotic cells was found. Quantitative histological analysis revealed that almost all apoptotic hepatocytes exhibiting chromatin condensation (early histological stage of apoptosis) as well as 70% of apoptotic bodies (ABs) contain the precursor form of TGF-$\beta$1. The negative response in 30% of ABs may be due to advanced intracellular degradation in later stages of the apoptotic process (Wyllie *et al.*, 1980; Bursch *et al.*, 1990). Thus most if not all apoptotic hepatocytes seem to contain the epitopes of anti-pre(266–278). The failure to detect mature TGF-$\beta$1 in the majority of apoptotic cells can probably be explained by its rapid degradation. The biological half-life of the pre-form is about 2 h, that of mature TGF-$\beta$1 is only 2 min. (Coffey *et al.*, 1987; Wakefield *et al.*, 1990). Thus, once mature TGF-$\beta$1 is formed, its rapid degradation should result in low levels in apoptotic cells that may be insufficient for its unequivocal immunocytochemical detection with the antibodies used.

Furthermore, we cannot rule out that anti-pre(266–278) cross-reacts with other molecules involved in apoptosis. However, we observed a colocalisation, albeit in only some apoptotic bodies, of responses with anti-pre(266–278) and anti-LC(1–30). A further line of evidence for the involvement of TGF-$\beta$1 in apoptosis is provided by the cell culture studies which show that both forms of TGF-$\beta$1 can induce apoptosis of hepatocytes. These observations support our hypothesis that our immunocytochemical findings indicate the presence of forms of TGF-$\beta$1 in apoptotic hepatocytes.

In the present study also histologically intact hepatocytes apparently preparing for apoptosis could be visualised with the anti-pre(266–278) antibody. The duration of this preparative, pre(266–278) TGF-β1 positive stage can be estimated by assuming that all positive hepatocytes are on the pathway to apoptosis and hence their incidence is proportional to the duration of this stage. Since this incidence was similar to that of AB which persist in a visible form for about 3 h (Bursch *et al.*, 1990) the presence of TGF-β1 in hepatocytes prone to apoptosis likewise may last approximately 3 h before chromatin condensation starts. Up to now this preparative stage has not been detectable. The use of (pre-)TGF-β1 antibodies as potential marker for pre-apoptotic cells may render studies on early events in apoptosis possible.

Necrotic cells occurring in the liver after carbon tetrachloride or after N-nitrosomorpholine were found to be negative for anti-pre(266–278) TGF-β1. However, in some apoptotic bodies and apparently intact hepatocytes pre-TGF-β1 was detected. Thus the corresponding epitopes may specifically occur in apoptotic cells. This observation may be of considerable interest in view of existing difficulties to discriminate between apoptosis and necrosis. Furthermore, our observations support previous reports that both necrosis and apoptosis may take place after toxic liver injury (Wyllie, 1987).

In an experiment with regressing rat uterus post partum we also observed a positive immunostaining of apoptotic bodies with anti-pre(266–278). These findings *in vivo* are consistent with recent observations *in vitro* on induction of apoptosis by TGF-β1 in primary endometrial cells (Rotello *et al.*, 1991). They show that the appearance of pre(266–278) TGF-β1 in apoptotic cells is not specific for hepatocytes, but may occur in other epithelia as well.

In addition to the immunocytochemical analysis, studies with primary hepatocyte cultures provided support for an involvement of TGF-β1 in apoptosis. It was found that (1) TGF-β1 can actively induce apoptosis; (2) TGF-β1 can induce apoptosis as an extrinsic factor; (3) the greater potency of mature TGF-β1 which was confirmed in four independent experiments suggests that this may be the active form. At present, we do not know the site of TGF-β1 synthesis and whether the increase of TGF-β1 protein is regulated at the transcriptional or post-transcriptional level. In previous studies non-parenchymal cells of the liver but not hepatocytes were found to express TGF-β1 (Carr *et al.*, 1989; Nagy *et al.*, 1989). The apparent occurrence of (pre-)TGF-β1 selectively in hepatocytes involved in apoptosis could be explained by uptake of TGF-β1 through the mannose 6-phosphate receptor (Jirtle *et al.*, 1991) or by specific synthesis. Studies on this question are in progress and will be published elsewhere. In any event, the present *in vivo* and *in vitro* data strongly suggest an involvement of TGF-β1 in the initiation of apoptosis in hyperplastic and (pre)neoplastic liver and possibly other epithelial tissues.

We would like to thank Drs A. Roberts, M. Sporn and K. Flanders for providing the TGF-β1 antibodies anti-LC(1–30) and anti-pre(266–278).

### References

AFTING, E.G. & ELCE, J.S. (1978). DNA in the rat uterus myometrium during pregnancy and postpartum involution. *Anal. Biochem.*, **86**, 90–99.

BURSCH, W., LAUER, B., TIMMERMAN-TROSIENER, I., BARTHEL, G., SCHUPPLER, J. & SCHULTE-HERMANN, R. (1984). Controlled cell death (apoptosis) of normal and putative preneoplastic cells in rat liver following withdrawl of tumor promoters. *Carcinogenesis*, **5**, 453–458.

BURSCH, W., TAPER, H.S., SOMER, M.P., MEYER, S., PUTZ, B. & SCHULTZE-HERMANN, R. (1989). Histochemical and biochemical studies on the effect of the prostacycline derivate Iloprost on $CCL_4$ induced lipid peroxidation in rat liver and its significance for hepatoprotection. *Hepatology*, **9**, 830–838.

BURSCH, W., PAFFE, S., PUTZ, B., BARTHEL, G. & SCHULTE-HERMANN, R. (1990). Determination of the length of the histological stages of apoptosis in normal liver and in altered hepatic foci of rats. *Carcinogenesis*, **11**, 847–853.

BURSCH, W., LIEHR, J.G., SIRBASKU, D., PUTZ, B., TAPER, H. & SCHULTE-HERMANN, R. (1991). Control of cell death (apoptosis) by diethylstilbestrol in an estrogen dependent kidney tumor. *Carcinogenesis*, **12**, 855–860.

CARR, B.I., HAYASHI, I., BRANUM, E.L. & MOSES, H.L. (1986). Inhibition of DNA synthesis in rat hepatocytes by platelet-derived TGF-β1. *Cancer Res.*, **46**, 2330–2334.

CARR, B.I., HUANG, T.H., ITAKURA, K., NOEL, M. & MARCEAU, N. (1989). TGF-beta gene transcription in normal and neoplastic liver growth. *J. Cell Biochem.*, **39**, 477–487.

COFFEY, R.J., KOST, L.J., LYONS, R.M., MOSES, H.L. & LARUSSO, N.F. (1987). Hepatic processing of transforming growth factor β in the rat. Uptake, metabolism, and biliary excretion. *J. Clin. Invest.*, **80**, 750–757.

COLETTA, A.A., WAKEFIELD, A.M., HOWELL, F.V., DANIELPOUR, D., BAUM, M. & SPORN, M.B. (1991). The growth inhibition of human breast cancer cells by a novel synthetic progestin involves the induction of transforming growth factor-β. *J. Clin. Invest.*, **87**, 277–283.

COLUMBANO, A., LEDDA-COLUMBANO, G.M., RAO, P.M., RAJALAKSHMI, S. & SARMA, D.S.R. (1984). Occurrence of cell death (apoptosis) in preneoplastic and neoplastic liver cells. *Am. J. Pathol.*, **116**, 441–446.

DUKE, R.C. & COHEN, J.J. (1986). Il-2 Addiction: withdrawl of growth factor activates a suicide program in dependent T-cells. *Lymphokine Res.*, **5**, 289–299.

FLANDERS, K.C., THOMPSON, N.L., CISSEL, D.S., VAN OBBERGHEN-SCHILLING, E., BAKER, C.C., KASS, M.E., ELLINGSWORTH, L.R., ROBERTS, A. & SPORN, M.B. (1989). Transforming growth factor-β1: Histochemical localization with antibodies to different epitopes. *J. Cell Biol.*, **108**, 653–660.

GARCEA, R., DAINO, L., PASCALE, R., SIMILE, M., PUDDU, M., FRASSETTO, S., COZZOLINO, P., SEDDAIU, M., GASPA, L. & FEO, F. (1989). Inhibition of promotion and persistent nodule growth by S-adenosyl-L-methionine in rat liver carcinogenesis: Role of remodelling and apoptosis. *Cancer Res.*, **49**, 1850–1856.

GENTRY, L.E., LIOUBIN, M.N., PURCHIO, A.F. & MARQUARDT, H. (1988). Molecular events in the processing of recombinant type 1 pre-pro-transforming growth factor beta to the mature polypeptide. *Mol. & Cell. Biol.*, **8**, 4162–4168.

HENDERSON, S., ROWE, M., GREGORY, C., CROOM-CARTER, D., WANG, F., LONGNECKER, R., KIEFF, E. & RICKINSON, A. (1991). Induction of bcl-2 expression by Epstein-Barr virus latent membrane protein 1 protects infected B cells from programmed cell death. *Cell*, **65**, 1107–1115.

JIRTLE, R. & MEYER, S.A. (1991). Liver tumour promotion: effect of phenobarbital on EGF and protein-kinase C signal transduction and transforming growth factor-betal expression. *Digest. Dis. Sci.*, **36**, 659–668.

JIRTLE, R.L., CARR, B.I. & SCOTT, C.D. (1991). Modulation of insulin-like growth factors-II/mannose 6-phosphate receptors and transforming growth factor-β1 during liver regeneration. *J. Biol. Chem.*, **266**, 22444–22450.

KERR, J.F.R., WYLLIE, A.H. & CURRIE, A.R. (1972). Apoptosis: a basic biological phenomenon with wide-ranging implications in tissue kinetics. *J. Cancer*, **26**, 239–257.

KOURY, M.J. & BONDURANT, M. (1990). Erythropoietin retards DNA breakdown and prevents programmed cell death in erythroid progenitors cells. *Science*, **248**, 378–381.

KYPRIANOU, N. & ISAACS, J.T. (1989). Expression of transforming growth factor in the rat ventral prostate during castration-induced programmed cell death. *Mol. Endocrinol.*, **3**, 1515–1522.

KYPRIANOU, N., ENGLISH, H.F. & ISAACS, J.T. (1990). Programmed cell death during regression of PC-82 human prostate cancer following androgen ablation. *Cancer Res.*, **50**, 3748–3753.

MIYAZONO, K., HELLMAN, U., WERNSTEDT, CH. & HELDIN, C.-H. (1988). Latent high molecular weight complex of transforming growth factor β1. *J. Biol. Chem.*, **263**, 6407–6415.

NAGY, P., EVARTS, R.P., MCMAHON, J.B. & THORGEISSON, S.S. (1989). Role of TGF-beta in normal differentiation and oncogenesis in rat liver. *Mol. Carcinogenesis*, **2**, 345–354.

NAKATSUKASA, H., NAGY, P., EVARTS, R.P. HSIA, C.-C., MARSDEN, E. & THORGEIRSSON, S.S. (1990). Cellular distribution of transforming growth factor β1 and procollagen types I, II and IV transcripts in carbon tetrachloride-induced rat liver fibrosis. J. Clin. Invest., **85**, 1833–1843.

NUNEZ, G., LONDON, L., HOCKENBERRY, D., ALEXANDER, M., MCKEARN, J.P. & KORSMEYER, S.J. (1990). Deregulated Bcl-2 gene experssion selectively prolongs survival of growth factor-deprived hemopoietic cell lines. *J. Immun.*, **144**, 3602–3610.

OBERHAMMER, F., BURSCH, W., PARZEFALL, W., BREIT, P., ERBER, E., STADLER, M. & SCHULTE-HERMANN, R. (1991). Effect of transforming growth factor β on cell death of cultured rat hepatocytes. *Cancer Res.*, **51**, 2478–2485.

OBERHAMMER, F., PAVELKA, M., SHARMA, S., TIEFENBACHER, R., PURCHIO, A.F., BURSCH, W. & SCHULTE-HERMANN, R. (1992). Induction of apoptosis in cultured hepatocytes and in regressing liver by transforming growth factor β. *PNAS*, **89**, 5408–5412.

ROTELLO, R.J., LIEBERMAN, R.C., PURCHIO, A.F. & GERSCHENSON, L.E. (1991). Coordinated regulation of apoptosis and cell proliferation by transforming growth factor β1 in cultured uterine epithelial cells. *Proc. Natl Acad. Sci. USA*, **88**, 3412–3415.

RUSSELL, W.E., COFFEY, R.J., QUELLETTE, A.J. & MOSES, H.L. (1988). TGF-β reversibly inhibits the early proliferative response to partial hepatectomy. *Proc. Natl Acad. Sci. USA*, **85**, 5126–5630.

SAVILL, J., DRANSFIELD, I., HOGG, N. & HASLETT, CH. (1990). Vitronectin receptor-mediated phagocytosis of cells undergoing apoptosis. *Nature*, **343**, 170–173.

SCHULTE-HERMANN, R., TIMMERMANN-TROSIENER, I., BARTHEL, G., BURSCH, W. (1990). DNA Synthesis, apoptosis and phenotypic expression as determinants of growth of altered foci in rat liver during phenobarbital promotion. *Cancer Res.*, **50**, 5127–5135.

STERNBERGER, L.A., HENDY, P.H., CUCULIES, I.I. & MEYER, H.G. (1970). The unlabeled antibody enzyme method of immunohistochemistry: preparation and properties of soluble antigen-antibody complex (horseradish peroxidase-anti-peroxidase and its use in identification of spirochaetes. *J. Histochem. Cytochem.*, **18**, 313–315.

SZENDE, B., SRKALOVIC, G., GROOT, K., LAPIS, K. & SCHALLY, A.V. (1990). Regression of nitrosamine-induced pancreatic cancers in hamsters treated with luteinizing hormone releasing hormone antagonists or agonists. *Cancer Res.*, **50**, 3716–3721.

THOMPSON, N.L., FLANDERS, K.C., SMITH, J.M., ELLINGWORTH, L.R., ROBERTS, A.B. AND SPORN, M.B. (1989). Expression of transforming growth factor β1 in specific cells and tissues of adult and neonatal mice. *J. Cell Biol.*, **108**, 661–669.

TRAUTH, B.C., KLAS, C., PETERS, A.M., MATZUKO, S., MÖLLER, P., FALK, W., DEBATIN, K.-M. & KRAMMER, P.H. (1989). Monoclonal antibody-mediated tumor regression by induction of apoptosis. *Science*, **245**, 301–305.

WAKEFIELD, L., WINOKUR, T.S., HOLLANDS, R.S., CHRISTOPHERSON, K., LEVINSON, A.D. & SPORN, M.B. (1990). Recombinant latent transforming growth factor β1 has a longer plasma half-life in rats than active transforming growth factor β1, and a different tissue distribution. *J. Clin. Invest.*, **86**, 1976–1984.

WILLIAMS, G.T., SMITH. CH.A., SPOONCER, E., DEXTER, M.T. & TAYLOR, D.R. (1990). Haemopoietic colony stimulating factors promote cell survival by suppressing apoptosis. *Nature*, **343**, 76–79.

WYLLIE, A.H. (1985). The biology of cell death in tumors. *Anticancer Res.*, **5**, 131–136.

WYLLIE, A.H. (1987). Apoptosis: cell death under homoestatic control. *Arch. Toxicol.*, Suppl. 11, 3–10.

WYLLIE, A.H., KERR, J.F.R. & CURRIE, A.R. (1980). Cell death: the significance of apoptosis. *Int. Rev. Cytol.*, **68**, 251–306.